# Rho GTPase-activating protein 10 (ARHGAP10/GRAF2) is a novel autoantibody target in patients with autoimmune encephalitis

**DOI:** 10.1007/s00415-022-11178-9

**Published:** 2022-05-27

**Authors:** Sven Jarius, Lars Komorowski, Jens U. Regula, Jürgen Haas, Stefanie Brakopp, Brigitte Wildemann

**Affiliations:** 1 Molecular Neuroimmunology Group, Department of Neurology, University of Heidelberg, Heidelberg, Germany; 2 Institute for Experimental Immunology, EUROIMMUN Medizinische Labordiagnostika AG, Lübeck, Germany; 3 Department of Neurology, University of Heidelberg, Heidelberg, Germany; 4 Department of Neurology, SRH Kurpfalzkrankenhaus Heidelberg, Heidelberg, Germany

**Keywords:** Cerebellar ataxia, Rho GTPase-activating protein 26 (ARHGAP26), Rho GTPase-activating protein 10 (ARHGAP10), Autoantibodies, Anti-Ca, Anti-Ca2, Antibodies, Immunoglobulin G (IgG), Autoimmune encephalitis, Limbic encephalitis, Polyneuropathy, Cognitive decline, Antigen, GTPase regulator associated with focal adhesion kinase (GRAF), GRAF2, Oligophrenin-like protein 1 (OPHN1L), Medusa head ataxia

## Abstract

**Background:**

In 2010, we described a novel immunoglobulin G (IgG) autoantibody (termed anti-Ca after the index case) targeting Rho GTPase-activating protein 26 (ARHGAP26, also termed GTPase regulator associated with focal adhesion kinase [GRAF], or oligophrenin-like protein 1 [OPHN1L]) in autoimmune cerebellar ataxia (ACA). Later, ARHGAP26-IgG/anti-Ca was reported in patients with limbic encephalitis/cognitive decline or peripheral neuropathy. In several of the reported cases, the syndrome was associated with cancer. ARHGAP10/GRAF2, which is expressed throughout the central nervous system, shares significant sequence homology with ARHGAP26/GRAF. Mutations in the ARHGAP10 gene have been linked to cognitive and psychiatric symptoms and schizophrenia.

**Objective:**

To assess whether ARHGAP26-IgG/anti-Ca co-reacts with ARHGAP10.

**Methods:**

Serological testing for ARHGAP10/GRAF2 autoantibodies by recombinant cell-based assays and isotype and IgG subclass analyses.

**Results:**

26/31 serum samples (84%) from 9/12 (75%) ARHGAP26-IgG/anti-Ca-positive patients and 4/6 ARHGAP26-IgG/anti-Ca-positive CSF samples from four patients were positive also for ARHGAP10-IgG. ARHGAP10-IgG (termed anti-Ca2) remained detectable in the long-term (up to 109 months) and belonged mainly to the complement-activating IgG1 subclass. Median ARHGAP26-IgG/anti-Ca and median ARHGAP10-IgG/anti-Ca2 serum titres were 1:3200 and 1:1000, respectively, with extraordinarily high titres in some samples (ARHGAP26-IgG/anti-Ca: up to 1:1000,000; ARHGAP10-IgG: up to 1:32,000). ARHGAP26/anti-Ca serum titres exceeded those of ARHGAP10-IgG in all samples but one. A subset of patients was positive also for ARHGAP10-IgM and ARHGAP10-IgA. CSF/serum ratios and antibody index calculation suggested intrathecal production of ARHGAP26-IgG/anti-Ca and anti-ARHGAP10. Of 101 control samples, 100 were completely negative for ARHGAP10-IgG; a single control sample bound weakly (1:10) to the ARHGAP10-transfected cells.

**Conclusions:**

We demonstrate that a substantial proportion of patients with ARHGAP26-IgG/anti-Ca-positive autoimmune encephalitis co-react with ARHGAP10. Further studies on the clinical and diagnostic implications of ARHGAP10-IgG/anti-Ca2 seropositivity in patients with autoimmune encephalitis are warranted.

**Supplementary Information:**

The online version contains supplementary material available at 10.1007/s00415-022-11178-9.

## Introduction

### Background

In 2010, we identified a novel high-titre serum reactivity (termed anti-Ca) targeting the Rho GTPase-activating protein 26 (ARHGAP26, also termed GTPase regulator associated with focal adhesion kinase [GRAF], or oligophrenin-like protein 1 [OPHN1L]) in a patient with autoimmune cerebellar ataxia (ACA) [11]. The patient had presented with rapidly progressive ACA leading to marked cerebellar atrophy and severe disability. The antibodies were present at high titres, were produced intrathecally, and belonged to the IgG1 subclass, suggesting that ARHGAP26-IgG/anti-Ca may be not only of diagnostic but also of pathogenetic impact.

ARHGAP26 is expressed predominantly in Purkinje cells (PC) in the cerebellum, but also by a subset of neurons in the hippocampus. Following up on our original report, we described five further ARHGAP26-IgG/anti-Ca-positive patients with ACA [2, 10, 28]. Two of these patients, as well as another patient without ACA, had signs of possible limbic encephalitis [13], indicating that the spectrum of neurological manifestations associated with ARHGAP26-IgG/anti-Ca may be broader than originally thought. In three further cases, isolated cognitive decline was noted [1]. In addition, 17 further ARHGAP26/anti-Ca-positive patients were reported by the Mayo Clinic in 2020, 15 of whom had subacute progressive cerebellar ataxia and 2 peripheral neuropathy [15], and several as yet unpublished additional cases were identified in our laboratories over the subsequent years. Considering that a substantial number of these cases were associated with cancer (including squamous cell carcinoma of the lung, ovarian cancer, prostate cancer, gastric adenocarcinoma, B-cell lymphoma and thymoma), ARHGAP26-IgG/anti-Ca-positive encephalitis is considered a facultative paraneoplastic neurological syndrome. Expression of ARHGAP26 at protein or RNA level has been shown for a multitude of solid tumours and cancer cell lines [24].

Several paralogues of ARHGAP26 have been reported. We were thus interested in whether ARHGAP26-IgG/anti-Ca may cross-react with other members of the ARHGAP family or related proteins. A database search revealed the Rho GTPase 10 (ARHGAP10/GRAF2) gene as a particularly important paralogue of ARHGAP26/GRAF [26]. Accordingly, some regions within the proteins coded by these two genes show significant sequence homology [26]. Animal data suggest that ARHGAP10 is widely expressed throughout the brain, including the cerebellum [14, 24]. Therefore, we hypothesised that cross-reactivity of ARHGAP26-IgG/anti-Ca may render ARHGAP10 an additional immune target in patients with ACA and, possibly, other forms of autoimmune encephalitis associated with ARHGAP26-IgG/anti-Ca seropositivity.

To explore this hypothesis, we developed a cell-based immunoassay employing recombinant ARHGAP10 as antigenic substrate and tested sera from both ARHGAP26-IgG/anti-Ca-positive patients and controls for autoantibodies to ARHGAP10.

We found that serum and CSF IgG from ARHGAP26-IgG/anti-Ca-positive patients indeed co-reacts with ARHGAP10 in a substantial proportion of cases, rendering ARHGAP10 a new target antigen in autoimmune encephalitis. We show that the antibodies belong to the complement-activating IgG1 subclass, with IgM and IgA antibodies being present in addition; are detectable already at disease onset; remain present over the entire course of disease; and are produced intrathecally. Our data suggest that additional autoimmunity to ARHGAP10 might contribute to the pathogenesis of neuroinflammation in a subgroup of ARHGAP26-IgG/anti-Ca-positive patients.

### Methods

#### Cell-based assay (CBA)

The coding DNA for human ARHGAP10 (NCBI Reference Sequence: NP_078881.3, synthetic gene, Eurofins Genomics, Germany) or ARHGAP26 (cDNA according to NCBI Accession BC068555, Source BioScience LifeSciences, Nottingham, UK), respectively, was transferred into the expression vector pTriEx-1 (Novagen). The proteins were transiently expressed in the human cell line HEK293. For the preparation of substrates for a recombinant cell-based indirect immunofluorescence assay (CBA), HEK293 were grown on sterile cover glasses, transfected, and allowed to express the recombinant proteins for 48 h. Cover glasses were washed with PBS, fixed with acetone for 10 min at room temperature, air-dried, cut into millimetre-sized fragments (biochips), and used as substrates in the CBA. ARHGAP10-transfected cells, ARHGAP26-transfected cells and mock-transfected cells were then incubated with patient serum diluted 1:10 in PBS. An FITC-labelled goat anti-human IgG secondary antibody was used to detect bound patient IgG (Euroimmun, Lübeck, Germany). Sera binding to ARHGAP10-transfected cells but not to control cells were considered positive. For evaluation of IgG subclasses, unconjugated sheep anti-human IgG antibodies specific for IgG subclasses 1 to 4 (The Binding Site, Schwetzingen, Germany) were substituted for the FITC-labelled goat anti-human IgG antibody, and AF568-labelled donkey anti-sheep IgG (Invitrogen; absorbed against human IgG) was used to detect the subclass-specific antibodies. Signal intensity at starting dilution (1:10) was assessed visually using a semiquantitative 5-point score already applied in previous studies (1 = very weak, 2 = weak, 3 = intermediate, 4 = strong, 5 = very strong fluorescence). Serum and CSF samples positive at 1:10 dilution were further titrated.

#### Antibody index (AI) calculation

Intrathecal synthesis of ARHGAP26-IgG/anti-Ca and ARHGAP10-IgG antibodies was assessed by calculation of the corresponding antibody indices: AI = QIgG [spec]/QIgG[total], if QIgG [total] < Qlim, and AI = QIgG [spec]/Qlim, if QIgG [total] > Qlim [16]. The upper reference range of QIgG [total], Qlim, was calculated according to Reiber’s formula to correct for possible underestimation of intrathecal specific synthesis due to possible blood‒CSF barrier disturbance [16]. AI values > 4 were considered indicative of intrathecal anti-Purkinje cell IgG production [17].

#### Western blotting (WB)

For the WB, mouse cerebellum lysate (sc-2403; Santa Cruz, TX, USA) was blotted on a Immobilon-P PVDF transfer membrane (0.45 µm) (Merck, Darmstadt, Germany), cut into strips and incubated with patient serum (1:20 dilution in PBS) and polyclonal rabbit antibodies to human ARHGAP26 (PTGLab, Manchester, UK) or ARHGAP10 (PTGLab), respectively. Binding of serum IgG was detected using a IRDye 800 CW-labelled goat anti-human IgG antibody (Euroimmun). An IRDye 680 RD-labelled goat antibody was used to detect bound rabbit antibodies to ARHGAP26 or ARHGAP10. An Odyssey™ fluorescence scanner (Licor) was then used to analyse the blots for antibody binding.

#### Tissue-based assay (TBA)

Unfixed 10 µm cryosections of rhesus monkey cerebellum (Euroimmun) were incubated with patient serum diluted in PBS. Binding of patient IgG was detected using a primate IgG-preadsorbed FITC-labelled anti-human IgG antibody (Euroimmun). PBS was used for washing the cells after each incubation step. Slides were then mounted with ProLongGold^®^ (Invitrogen) containing DAPI and analysed using a Nikon Ni-E fluorescence microscope (Nikon Imaging Center, Heidelberg, Germany).

#### Samples

Thirty-one serum and 6 CSF samples from 12 ARHGAP26-IgG/anti-Ca-positive patients with either autoimmune cerebellar ataxia and/or signs of limbic encephalitis/cognitive decline were available for testing. In parallel, 101 control samples were tested, including 30 × serum and 2 × CSF from 31 patients with ACA of unknown cause, 21 × serum from 21 patients with other autoantibody-associated CNS disorders (Homer-3-IgG, CARPVIII-IgG, ITPR1-IgG, mGluR-delta2-IgG, PKC-gamma-IgG, anti-Hu-IgG, anti-IgG, anti-Tr/DNER, anti-amphiphysin, anti-GAD65-, anti-CV2/CMRP5, CASPR2-IgG, LGI1-IgG, NMDAR-IgG, AMPAR-IgG, GABABR-IgG, Sox1-IgG, Zic4-IgG, DPPX-IgG, IgLON5-IgG, GFAP-IgG), 20 × serum and 11 × CSF from 31 patients with multiple sclerosis (MS) (16 × actively relapsing, 15 × chronic progressive course), 7 × CSF from 7 patients with headache but otherwise unremarkable clinical and radiological neurological workup, and 10 × serum from 10 healthy controls [HC]. Samples were analysed as part of a larger project on the differential diagnosis of neuroimmunological diseases. The study was approved by the institutional review board of the University of Heidelberg. Patients gave written informed consent or analysis was performed in an anonymized fashion as required by the institutional review board of the University of Heidelberg. No samples were obtained specifically for the purpose of this study. Samples were stored at − 80 °C prior to testing.

## Results

### CBA

**Fig. 1:**
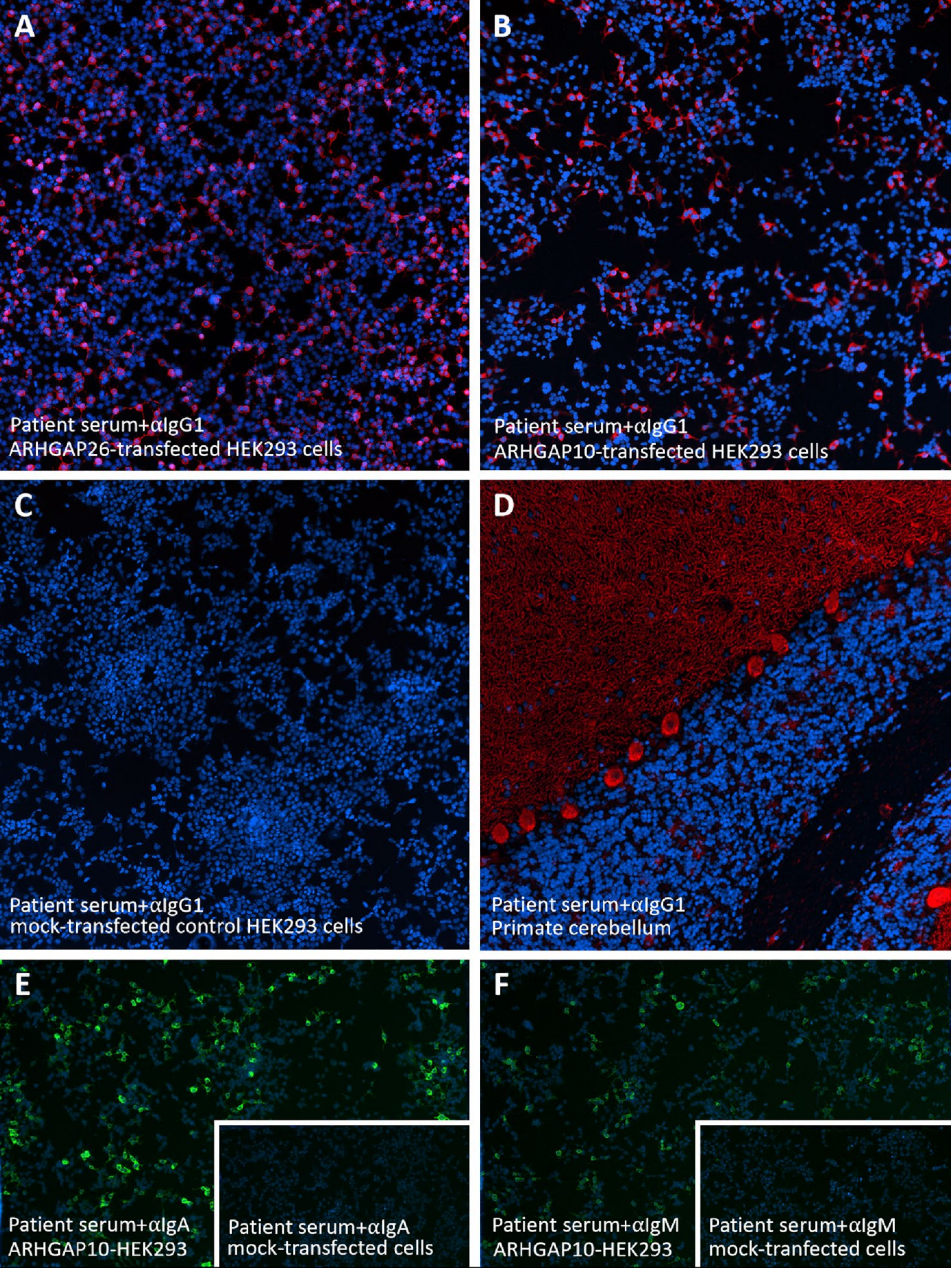
Binding of serum IgG1 (**A**-**D**), IgA (**E**) and IgM (**F**) from a patient with ACA to both ARHGAP26-transfected (**A**) and ARHGAP10-transfected HEK293 cells (**B**, **E**–**F**) and to primate cerebellum tissue sections (**D**) but not to mock-transfected HEK293 cells (**C**, **E** inset, **F** inset). Red (AF568) indicates bound IgG1 in (**A**-**D**); green (FITC) indicates bound IgA or IgM in (**E**–**F**); blue indicates nuclear staining by DAPI

Eighteen of 18 serum samples and 2 of 2 CSF samples from the ARHGAP26-IgG/anti-Ca index patient originally reported in 2008 [11], obtained over a period of 109 months (with the earliest sample taken just 8 days after onset of symptoms), showed IgG binding to both the ARHGAP26-transfected and the ARHGAP10-transfected HEK293 cells (Fig. 1). As previously found with ARHGAP26 [11], acetone fixation resulted in much stronger binding of patient IgG to ARHGAP10 (serum: signal intensity 4–5 at 1:10 screening dilution; CSF: signal intensity 5 when tested undiluted) than did formalin fixation (serum: signal intensity 1 at 1:10 screening dilution; CSF: signal intensity 0 when tested undiluted).

Subsequently, we tested another 13 serum samples from 11 further previously identified ARHGAP26-IgG/anti-Ca-positive patients and 4 additional CSF samples from 3 patients. Of these, 4 serum samples from 4 patients showed distinct binding to the acetone-fixed ARHGAP10-transfected HEK293 cells (signal intensities 5, 5, 3 and 2 at 1:10 screening dilution, respectively) and 2 CSF samples from 1 patient (signal intensity 2 and 5, respectively, when tested undiluted); 4 further serum samples from 4 additional patients showed faint staining; and 5 ARHGAP26-IgG/anti-Ca-positive sera from 4 patients and 2 CSF samples from 2 patients bound exclusively to ARHGAP26 but not to ARHGAP10.

In summary, 26/31 sera (84%) from 9/12 (75%) ARHGAP26-IgG/anti-Ca-positive patients were positive for ARHGAP10-IgG, including all follow-up sera from the ARHGAP26-IgG/anti-Ca-positive index patient and 4/6 ARHGAP26-IgG/anti-Ca-positive CSF samples from 4 patients. Similarly, the ARHGAP10-IgG antibody status did not change in follow-up CSF samples (2 × positive in each case) from the only 2 ARHGAP26-IgG/anti-Ca patients in whom repeat LP was performed.

Of the 101 control samples, 100 were negative for antibodies to ARHGAP10 or ARHGAP26, irrespective of whether acetone or formalin was used as fixative. One control patient with ACA attributed to anti-protein kinase C gamma-related autoimmunity reacted very weakly with the acetone-fixed (but not with the formalin-fixed) ARHGAP10-transfected cells (1:10 positive; 1:32 equivocal staining only); however, mild staining also of acetone-fixed mock-transfected control cells was seen, leaving the possibility of a non-specific reaction. None of the ARHGAP10-IgG-positive samples and none of the remaining control samples bound to the mock-transfected HEK293 control cells, again irrespective of fixative.

### Serum titration

The index patient’s first available sample, taken shortly after onset, was positive for ARHGAP26-IgG/anti-Ca at a serum titre of 1:10,000 and for ARHGAP10-IgG at a serum titre or 1:1000 as determined by means of ARHGAP26- and ARHGAP10-specific CBAs, respectively. Two follow-up samples from the same patient, obtained 5 and 7 months later, were positive at a titre of 1:10,000 (ARHGAP26-IgG/anti-Ca) and 1:1000 or 1:3200 (ARHGAP10-IgG), respectively (Table 1). Both antibodies were still detectable in the last two samples available from the index patient, taken 109 months after onset, albeit at slightly lower titres (ARHGAP26: 1:3200; ARHGAP10: 1:1000).

**Table 1:** ARHGAP10-IgG/anti-Ca2 in the serum and CSF of 12 ARHGAP26-IgG/anti-Ca-positive patients

Patient	Sample	Days since onset	TBA Cerebellum	CBA ARHGAP26	End titres	CBA ARHGAP10	End titres
			Fluorescence signal intensity at screening dilution (1:10)	Fluorescence signal intensity at screening dilution (1:10)		Fluorescence signal intensity at screening dilution (1:10)	
*a. Serum samples*							
1	1	5	+ +	+ + + + +	1:10,000	+ + + + +	1:1000
	2	167	+ + +	+ + + + +	nd	+ + + + +	nd
	3	173	+ + +	+ + + +	1:10,000	+ + + +	1:1000
	4	211	+ + +	+ + + + +	1:10,000	+ + + + +	1:3200
	5	237	+ + +	+ + + + +	nd	+ + + + +	nd
	6	375	+ + +	+ + + + +	nd	+ + + + +	nd
	7	557	+ + +	+ + + + +	nd	+ + + + +	nd
	8	1160	+ + +	+ + + +	nd	+ + + +	nd
	9	1252	+ + +	+ + + + +	nd	+ + + + +	nd
	10	1357	+ + +	+ + + + +	nd	+ + + + +	nd
	11	1469	+ + +	+ + + + +	nd	+ + + + +	nd
	12	1671	+ + +	+ + + + +	nd	+ + + + +	nd
	13	1959	+ + +	+ + + + +	nd	+ + + + +	nd
	14	2142	+ + +	+ + + + +	nd	+ + + + +	nd
	15	2330	+ + +	+ + + + +	nd	+ + + + +	nd
	16	2889	+ + +	+ + + + +	nd	+ + + + +	nd
	17	3246	+ + +	+ + + + +	1:3200	+ + + + +	1:1000
	18	3352	+ + +	+ + + +	1:3200	+ + +	1:1000
2	1	nd	+ + +	+ + +	1:1000	+	1:32
3	1	nd	+ + + +	+ + + +	1:320,000	+ + +	1:32,000
4	1	nd	+ + +	+ + + + +	1:1,000,000	+ + + + +	1:32,000
5	1	nd	+ + +	+ + + + +	1:1,000,000	+ + + + +	1:32,000
6	1	nd	+	+ +	1:320	+	1:10
7	1	nd	+ +	+ +	1:20	+	1:10
8	1	nd	+ + + +	+ +	1:320	+ +	1:320
9	1	nd^2^	+ +	+ +	1:100	+	1:10
	2	nd^2^	+ +	+ +	1:100	–	–
	3	nd^2^	–	+ + +	1:100	–	–
10	1	nd	+ + +	+ + +	1:3200	–	–
11	1	nd	+	+ +	1:32	–	–
12	1	nd	+ + + +	+ + + +	1:3200	+ +	1:100
*b. CSF samples*							
1	1	5	+ + + +	+ + + + +	1:2000	+ + + +	1:150
	2	167	+ + + +	+ + + + +	nd	+ + + +	nd
2	1	nd^3^	+ + +	+ + + +	1:140	+ +	1:10
	2	nd^3^	+ +	+ + + +	1:16	+ +	1:2
6	1	nd	–	+	1:1	–	–
10	1	nd	nd^1^	+ +	1:4	–	–

In addition, 101 control samples (32 × cerebellar ataxia of suspected autoimmune aetiology; 21 x other autoantibody-associated CNS disorders; 31 × multiple sclerosis; 7 × headache; 10 × healthy controls) were tested, including 81 serum samples and 20 CSF samples, 100 of which were negative for ARHGAP10-IgG/anti-Ca2 (1 control serum sample mildly positive: 1:10 positive; 1:32 equivocally positive); see Methods and Results for details.

^1^Insufficient material available

^2^Obtained over a period of 8 months; time since onset of neurological symptoms unknown

^3^Obtained over a period of 14 days; time since onset of neurological symptoms unknown.

*nd* = not done/no data

Serum samples from three further patients showed extraordinarily high CBA titres, ranging between 1:100,000 and 1:1,000,000 for ARHGAP26-IgG/anti-Ca and between 1:32,000 and 1:100,000 for ARHGAP10-IgG. In the remaining 6 patients, ARHGAP26-IgG/anti-Ca titres ranged between 1:10 and 1:1000 and ARHGAP10-IgG titres between 1:10 and 1:320.

If all samples are considered, the median ARHGAP26-IgG/anti-Ca titre was 1:3200 and the median ARHGAP10-IgG titre 1:1000. In all double-positive cases, the ARHGAP26-IgG/anti-Ca serum titres were higher than the ARHGAP10-IgG titres, except for one patient in whom titres were low (1:320) and did not differ between the two antibodies. ARHGAP26-IgG/anti-Ca titres were low in 3 of the 4 ARHGAP10-IgG-negative serum samples (1 × 1:32, 2 × 1:100, 1 × 1:3200) (Table 1).

### CSF titration

An amount of CSF sufficient for titration was available from two patients. In the index patient, an ARHGAP26-IgG/anti-Ca CSF titre of 1:1600 was found in the CBA and an ARHGAP10-IgG CSF titre of 1:150. In a second patient, the CSF titres for ARHGAP26-IgG/anti-Ca and ARHGAP10-IgG were 1:40 and 1:10, respectively. CSF ARHGAP26-IgG/anti-Ca titres were rather low (1:4 and 1:1, respectively) in the two CSF ARHGAP10-IgG-negative patients; one of these two patients was weakly positive for serum ARHGAP10 at the time of lumbar puncture (Table 1).

### Correlation of ARHGAP26-IgG/anti-Ca and ARHGAP10-IgG titres

**Fig. 2:**
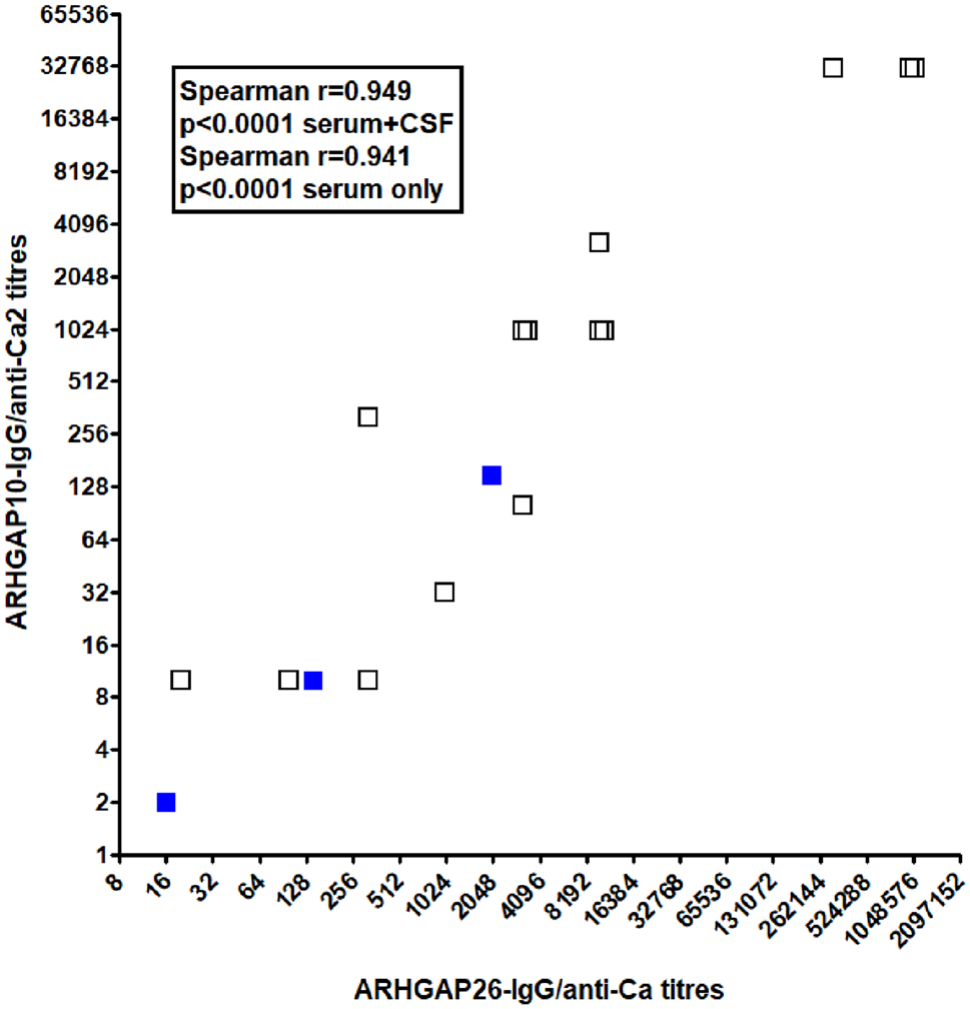
Strong correlation of ARHGAP10-IgG serum and CSF titres with ARHGAP26-IgG (anti-Ca) serum and CSF titres (*N* = 13 serum [black squares] and 3 CSF [blue squares] samples with available end titres), as determined by means of cell-based assays. Samples yielding identical titres are shown slightly set-off (double-square). Note the use of a logarithmic (log2) scale

ARHGAP10-IgG titres were strongly correlated with ARHGAP26-IgG/anti-Ca titres (*N* = 17 samples; *p* < 0.0001) (Fig. 2). The median ratio of ARHGAP26-IgG/anti-Ca titres to ARHGAP10 titres was 10 (range 1–32).

### Intrathecal synthesis

In both of the patients with available data, the respective CSF/serum ratios suggested intrathecal synthesis of both ARHGAP26-IgG/anti-Ca (index patient: ratio 1:2000/1:10,000 = 0.2; patient #2: ratio 1:40/1:1000 = 0.04) and ARHGAP10-IgG (index patient: ratio 1:100/1:1000 = 0.1; patient #2: ratio 1:10/1:32 = 0.3125) when compared with the normal average total IgG CSF/serum ratio of ~ 0.002. Albumin CSF and serum total IgG and total albumin levels were available only for the index patient due to anonymisation. In this patient, the ARHGAP26-IgG/anti-Ca-specific AI was 31.1 and the ARHGAP10-IgG-specific AI was 23.3 (cut-off: 4), demonstrating intrathecal synthesis of both antibodies based on CBA titres (a positive AI for ARHGAP26-IgG/anti-Ca had also been found in the same patient based on titres assessed by immunohistochemistry [11]). The patient was also positive for CSF-restricted total-IgG oligoclonal bands, and quantitative evidence for total-IgG intrathecal synthesis was found (intrathecally produced IgG fraction: 24% [11]).

### TBA

All but one of the serum samples positive for ARHGAP26-IgG/anti-Ca in the CBA showed the typical staining pattern associated with this antibody [11] when tested at screening dilution (1:10). However, signal intensities differed substantially (Table 1). While staining of the molecular layer was seen with all TBA-positive samples, additional staining of the PC somata was found only with high-titre samples (not shown). The only TBA-negative serum sample was obtained from a patient who was previously positive in the TBA twice and was positive for ARHGAP26-IgG/anti-Ca in the CBA only at low titre (1:100). No distinct differences in cerebellar binding patterns were observed between patients positive only for ARHGAP26-IgG/anti-Ca and patients positive for both antibody reactivities.

### Isotype analysis

Five serum samples from three patients were used for exemplary isotype analyses (Fig. 2). Sample 1 (from the ARHGAP26-IgG/anti-Ca- and ARHGAP10-IgG-positive index patient [11]) showed strong IgA (signal intensity 5/5) and IgM (4/5) reactivity to ARHGAP10 in the CBA, while (in accordance with previous analyses in the same patient [11]) only weak IgA reactivity and no IgM reactivity to ARHGAP26 was notable. By contrast, sample 2, obtained from another ARHGAP26-IgG/anti-Ca- and ARHGAP10-IgG-positive patient, showed strong IgA and IgM reactivity to both ARHGAP10 (both 3/5) and ARHGAP26 (5/5 and 4/5). Finally, three serum samples from an ARHGAP10-IgG-negative (but ARHGAP26-IgG/anti-Ca-positive) patient were also negative for ARHGAP10-IgA and ARHGAP10-IgM. No IgA or IgM reactivity was seen with an ARHGAP10-IgG-negative HC sample either.

### IgG subclass analysis

The ARHGAP26-IgG/anti-Ca-positive index patient [11] showed strong IgG1 and IgG2 serum reactivity and weak IgG3 and IgG4 serum reactivity to ARHGAP10 (signal intensity: IgG1 > IgG2 > IgG3 = IgG4) and strong IgG1, IgG2, IgG3 and IgG4 reactivity to ARHGAP26 (IgG1 > IgG2 = IgG4 > IgG3) in the CBA (Fig. 2). A CSF sample from the same patient was positive for both ARHGAP10-IgG1 and ARHGAP26-IgG1. The typical medusa head-like staining pattern was caused mainly by IgG1 autoantibodies also in the TBA (Fig. 2). In a second ARHGAP26-IgG/anti-Ca- and ARHGAP10-IgG-positive patient tested, also mainly IgG1 antibodies to ARHGAP10 were found (CBA: IgG1 > IgG2 > IgG3 = IgG4; TBA: IgG1 > IgG2 = IgG4 > IgG3), while an HC sample showed no IgG1, IgG2, IgG3 or IgG4 binding at all.

### Western blot

**Fig. 3:**
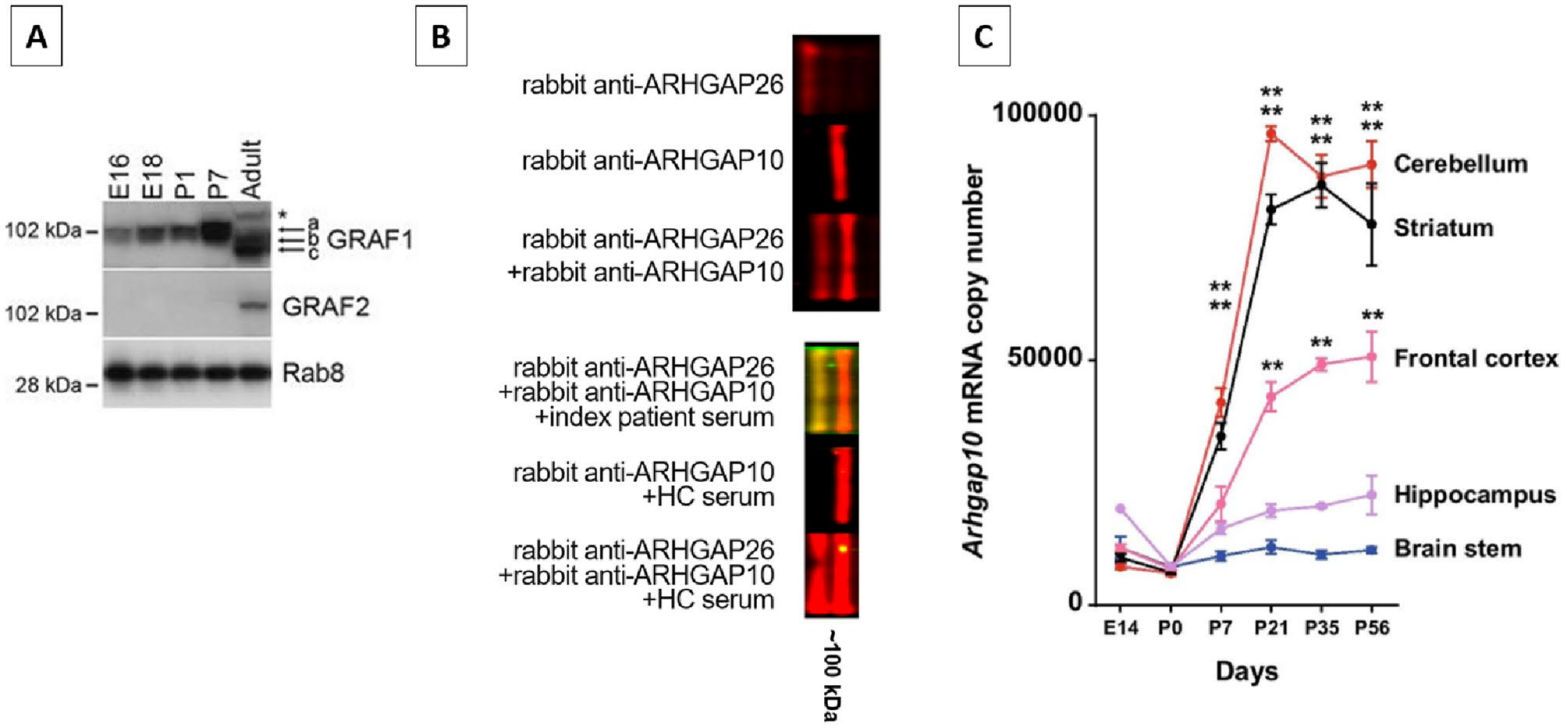
Demonstration of ARHGAP26 (GRAF1) and ARHGAP10 (GRAF2) and Arhgap10 mRNA expression in mouse brain and cerebellum. Panel **A** (reproduced from Safa Lucken-Ardjomande Häsler et al., GRAF1a is a brain-specific protein that promotes lipid droplet clustering and growth, and is enriched at lipid droplet junctions, J Cell Sci 2014, Fig. 1, under the terms of the Creative Commons Attribution License [http://creativecommons.org/licenses/by/3.0]) shows presence of the two antigens in mouse brain extracts from E16 and E18 embryos, P1 and P7 neonates, and from an adult, and demonstrates the developmentally regulated expression of both of the two proteins. Equal loading was verified on the same blot with an anti-Rab8 antibody. **B** Upper panel: ARHGAP10 and ARHGAP26 expression in mouse cerebellum extract as demonstrated by use of two rabbit antibodies (binding shown in red). Lower panel: While non-denatured ARHGAP10 expressed in HEK293 cells was clearly recognised by the patient’s serum IgG as shown in Fig. 1, no clear binding of the same patient’s IgG to ARHGAP10 (but to ARHGAP26) was seen in the Western blot, suggesting that the epitope detected by anti-ARHGAP10 might be sensitive to denaturing conditions. Red indicates binding of the two rabbit antibodies; green, binding of patient IgG; yellow, overlap of patient IgG and the respective rabbit antibodies. **C** Developmental changes in Arhgap10 mRNA levels in various brain regions in C57BL/6 J mice (*n* = 3 mice in each time point) (reproduced from Kazuhiro Hada et al. Mice carrying a schizophrenia-associated mutation of the Arhgap10 gene are vulnerable to the effects of methamphetamine treatment on cognitive function: association with morphological abnormalities in striatal neurons. Mol Brain 2021, Fig. 1, under the terms of the Creative Commons Attribution License [http://creativecommons.org/licenses/by/4.0])

Mouse cerebellum lysates were used to confirm the presence of ARHGAP10 in the cerebellum, as indicated by binding of a commercial antibody against ARHGAP10 in a WB assay (Fig. 3). Of note, the WB was not sensitive enough to detect ARHGAP10-IgG in the ARHGAP26-IgG/anti-Ca- and ARHGAP10-IgG-positive index patient’s serum (no other samples tested due to lack of material), suggesting that the patient’s autoantibodies may be specific for epitopes sensitive to the denaturing conditions during WB and may differ from the epitope recognised by the commercial anti-ARHGAP10 antibody.

## Discussion

Here we demonstrate that the majority of ARHGAP26/GRAF-IgG-positive patients with autoimmune encephalitis have antibodies that co-react with ARHGAP10/GRAF2, a protein expressed throughout the CNS.

ARHGAP10/GRAF2 shows the highest percentage of sequence matching of any paralogue of the ARHGAP26/GRAF gene [9], and substantial homology between the two proteins exists (Supplementary Fig. 1). Cross-reactivity of anti-neuronal antibodies with other, homologous antigens has been previously shown. For example, anti-Yo antibodies as detected by immunohistochemistry have been reported to bind to CDR2 (cerebellar degeneration-related 2; less commonly termed CDR62); to CDR3 [30], a protein similar to CDR2; and, in 85% of cases, to CDR2L (cerebellar degeneration-related 2-like) [12]. Similarly, anti-Ma-positive sera bind to PNMA1 and PNMA2 [8].

ARHGAP10/GRAF2 (not to be mixed up with ARHGAP21, which has been confusingly referred to as ARHGAP10 in a few publications) is, just like ARHGAP26, a GTPase activator for the small GTPases RhoA and Cdc42. Developmental data suggest that ARHGAP10 is present at protein level only in the adult mouse brain (at least at levels detectable by standard Western blotting), not in the embryonic state and not on postnatal day 7 (Fig. 3A) [14]. We confirmed its presence in adult mouse cerebellum by means of WB analysis (Fig. 3B). At RNA level, ARHGAP10 expression is detectable in most regions of the CNS both in mice [6], with highest levels observed in the cerebellum from day 27 onwards (Fig. 3C), and in humans [24]. Recently, a significant association between schizophrenia and exonic copy-number variations in the ARHGAP10 gene in Japanese patients has been reported [6, 20], constituting for the first time a link between ARHGAP10 and neurological disease. Symptoms present in the affected patients included cognitive decline and psychiatric symptoms requiring hospitalisation. Moreover, an association of ARHGAP10 mutations with morphological abnormality of neurons (e.g. loss of neural polarity, impaired neurite elongation, significantly reduced spine density) as well as cognitive, emotional and behavioural abnormalities has been observed in animal models [6, 20]. By regulating RhoA, ARHGAP10 might also affect GABA-A receptor endocytosis and degradation [18] and dopamine transporter internalisation [29], thereby potentially modulating neurotransmission.

The pathophysiological and clinical significance of the autoimmune response to ARHGAP10 in our patient still needs to be elucidated. It is conceivable that a simultaneous autoimmune reaction against two antigens is associated with a more severe disease course or with differences in lesion sites and clinical presentation, based on potential differences in ARHGAP26 and ARHGAP10 expression patterns within the nervous system. In fact, some previously reported ARHGAP26-IgG-positive patients with ACA developed symptoms suggesting additional extracerebellar CNS damage as well as peripheral nerve involvement [1, 2, 10, 13, 15, 28].

Future studies should also evaluate whether the presence or absence of additional reactivity to AHRGAP10 in patients with ARHGAP26-IgG/anti-Ca may be associated with clinical presentation, differences in long-term prognosis or a paraneoplastic aetiology and whether the serum and/or CSF levels or ARHGAP26-IgG/anti-Ca and ARHGAP10-IgG correlate with disease severity. Not enough clinical and follow-up data were available to address these questions in the present study due to anonymisation. Of note, however, ARHGAP10 is also expressed outside of the cerebellum, and autoimmunity to ARHGAP10 may thus explain the occurrence of extracerebellar symptoms in ARHGAP26-IgG/anti-Ca-positive patients.

While ARHGAP26-IgG/anti-Ca was initially identified in a patient with ACA, this patient had additional symptoms compatible with extracerebellar involvement (severe depression, restlessness, and anxiety). In fact, most previously reported ARHGAP26-IgG-positive patients had also signs suggestive of limbic involvement. This included cases of ACA associated with flattened affect, cognitive decline, working memory deficits, compromised verbal learning and recall, attention deficits, slowed information processing, interference difficulty, and reduced spatial recognition [2]; ACA associated with anterograde amnesia, attention deficits, disturbed word fluency, deficits of working and anterograde verbal memory, emotional instability, agitation, and depression; gait imbalance associated with progressive memory decline with deficits in language, abstraction, verbal memory, and orientation; cognitive impairment with deficits in short-term memory, attention, and executive function [1], and personality and behavioural changes, recurrent psychotic symptoms, suicidal thoughts, mutism, and apathy, without ACA [13]. Two further patients had ACA with pseudobulbar affect, associated with cognitive decline in one [15]. Interestingly, some patients had symptoms compatible with additional brainstem involvement (ACA with dysarthria, nausea/vomiting, and/or symptomatic hyperekplexia) [10, 11]. Detailed clinical data on the index patient (patient #1 in Table 1) can be found in reference [11].

Interestingly, by retrospectively testing 18 consecutive serum and CSF samples from a single ARHGAP26-IgG/anti-Ca-positive patient, we were able to demonstrate that reactivity to ARHGAP10 was present from the onset of disease. This indicates that ARHGAP10 did not result from epitope spreading occurring later in the disease course. On the other hand, clinical manifest autoimmune encephalitis is sometimes preceded by subclinical pre-stages. Therefore, epitope spreading could have occurred already in an earlier stage of disease and can thus not be fully ruled out.

In four samples from three ARHGAP26-IgG/anti-Ca-positive patients no antibodies to ARHGAP10 were detectable. This could indicate that ARHGAP26-IgG/anti-Ca targets exclusively ARHGAP26/GRAF-specific epitopes in some cases, and might reflect differences in epitope spreading among patients. However, this finding might also reflect differences in assay sensitivity, e.g. caused by differences in antigen expression levels of the transfected cells, or in antibody affinity, resulting in a positive anti-ARHGAP10 signal only if ARHGAP26-IgG/anti-Ca titres are sufficiently high: Firstly, ARHGAP26-IgG/anti-Ca were mostly low in the ARHGAP10-IgG-negative patients; secondly, ARHGAP10 levels were significantly and strongly correlated with ARHGAP26-IgG/anti-Ca levels; thirdly, in one of the three patients, ARHGAP10-IgG was negative on two occasions and weakly positive in a third sample.

One patient with anti-PKC-gamma-IgG-positive ACA co-reacted mildly with the acetone-fixed ARHGAP10-transfected cells. This could indicate the presence of two ACA-related antibodies in this patient. However, considering the low titre (1:10), a non-specific reaction cannot be excluded. One hundred further control samples, including 83 from patients with neuroimmunological disorders, were all negative for ARHGAP10.

It is a potential limitation that no data on the treatment status at the time of blood sampling was known due to anonymisation of the disease control samples. We can therefore not completely rule out that treatment played a role. Notably, however, ARHGAP26-IgG and ARHGAP10-IgG remained detectable in the index patient over many years despite intense immunosuppressive treatment with cyclophosphamide and even under combined treatment with methotrexate, leflunomide and six-monthly rituximab infusions, rendering major treatment effects unlikely. Moreover, ARHGAP10-IgG was negative in the HC and the headache control subgroup.

Although some evidence for a pathogenetic impact of autoantibodies to intracellular antigens exists (e.g. for anti-amphiphysin [3, 5, 22, 27], anti-Yo [19], anti-recoverin [21] and anti-GAD65 [4, 7]), it has been proposed that in autoimmune disorders targeting intracellular antigens, T cell-mediated rather than antibody-mediated pathomechanisms may dominate. It is therefore of note that, different from ARHGAP26/GRAF, membrane localisation has been predicted for ARHGAP10/GRAF2 in addition to cytoplasmic and perinuclear localisation, with association to cell membrane being dependent on the PH domain [26]. On the other hand, no membrane localisation of ARHGAP10 has been found in a number of cell lines investigated for the Human Protein Atlas project [23]. Assessing the pathogenetic impact of ARHGAP10-IgG antibodies in passive transfer animal experiments is hampered by the fact that all patients identified in this study were also positive for ARHGAP26-IgG/anti-Ca.

From a diagnostic point of view, it will be of interest to evaluate in future studies whether test sensitivity can be improved by employing cells transfected with both antigens.

## Conclusions

In summary, we have identified ARHGAP10 as a new autoantigen in patients with ARHGAP26-IgG/anti-Ca-positive autoimmune encephalitis. Our findings may either reflect cross-reactivity of ARHGAP26-IgG/anti-Ca with ARHGAP10 – as suggested by high sequence homology between the two proteins and the fact that all ARHGAP10-IgG/anti-Ca2-positive patients were positive also for ARHGAP26-IgG/anti-Ca in the present cohort – or, alternatively, indicate the presence of two distinct antibody reactivities in these patients. However, given the strong correlation of ARHGAP10-IgG/anti-Ca2 titres with ARHGAP26-IgG/anti-Ca titres, the former hypothesis seems much more likely. The intrathecal origin of ARHGAP10-IgG/anti-Ca2, the fact that the antibodies belonged mainly to the complement-activating IgG1 subclass, and the predicted membrane location would be compatible with a direct pathogenic role of the antibody. Further studies are warranted to define the frequency, specificity and pathophysiological relevance of ARHGAP10/anti-Ca2 antibodies in larger cohorts of patients with suspected autoimmune encephalitis and controls.

## Supplementary Information

Below is the link to the electronic supplementary material.

Supplementary file1 (DOCX 123 KB)

## Abbreviations

ACA: Autoimmune cerebellar ataxia
AI: Antibody index
ARHGAP26: Rho GTPase-activating protein 26
CBA: Cell-based assay
CDT: Clock-drawing test
CSF: Cerebrospinal fluid
FITC: Fluorescein isothiocyanate
GAD65: Glutamic acid decarboxylase, 65-kD isoform
GRAF: GTPase regulator associated with focal adhesion kinase
HEK293: Human embryonic kidney 293 cells
IgG/M/A: Immunoglobulin G/M/A
ITPR1: Inositol 1,4,5-trisphosphate receptor 1
IVIG: Intravenous immunoglobulins
IVMP: Intravenous methylprednisolone
MMSE: Mini-mental status examination
NMDAR: N-methyl D-aspartate receptors
OCB: Oligoclonal bands
OPHN1L: Oligophrenin-like protein 1
PC: Purkinje cells
PLEX: Plasma exchange
TBA: Tissue-based assay
