# Supplementary figures and images for: Rho GTPase-activating protein 10 (ARHGAP10/GRAF2) is a novel autoantibody target in patients with autoimmune encephalitis
Source: J Neurol. 2022 May 27;269(10):5420–30. doi: 10.1007/s00415-022-11178-9 (PMC9468106; doi:10.1007/s00415-022-11178-9)

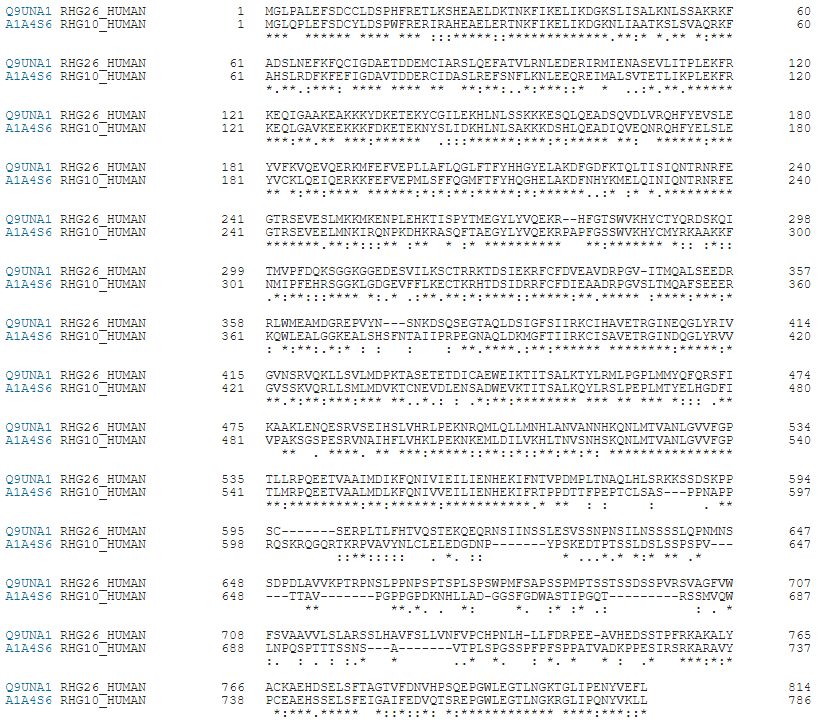


Supplementary figure 1.

Supplement: Supplementary file 1 — Supplementary file1 (DOCX 123 KB) [file 415_2022_11178_MOESM1_ESM.docx]
